# Dr. Simeon Holgate Owen

**Published:** 1909-06-05

**Authors:** 


### Obituary.

Dr. Simeon Holgate Owen, M.D.(Q.U.I.), M.R.C.P. (Lond.), died recently at Manchester at the age of 64. He was consulting physician to the Manchester Northern Hospital for Women and Children, and ex-president of the Manchester Clinical Society. He qualified at the Royal College of Surgeons and the London Society of Apothecaries in 1869, and held appointments for some years at the Manchester Royal Infirmary. Dr. Owen was the author of "Fevers : Their Nature and Prevention" and "Work and Recreation in their Relation to Health."

